# Salivary Biomarkers for Alzheimer’s Disease: A Systematic Review with Meta-Analysis

**DOI:** 10.3390/ijms25021168

**Published:** 2024-01-18

**Authors:** Kacper Nijakowski, Wojciech Owecki, Jakub Jankowski, Anna Surdacka

**Affiliations:** 1 Department of Conservative Dentistry and Endodontics, Poznan University of Medical Sciences, 60-812 Poznan, Poland; annasurd@ump.edu.pl; 2 Student’s Scientific Group in Department of Conservative Dentistry and Endodontics, Poznan University of Medical Sciences, 60-812 Poznan, Polandjjankowski41@wp.pl (J.J.)

**Keywords:** neurodegenerative diseases, Alzheimer’s Disease, saliva, biomarkers, beta-amyloid, tau, lactoferrin

## Abstract

Alzheimer’s Disease (AD) is the most common neurodegenerative disease which manifests with progressive cognitive impairment, leading to dementia. Considering the noninvasive collection of saliva, we designed the systematic review to answer the question “Are salivary biomarkers reliable for the diagnosis of Alzheimer’s Disease?” Following the inclusion and exclusion criteria, 30 studies were included in this systematic review (according to the PRISMA statement guidelines). Potential biomarkers include mainly proteins, metabolites and even miRNAs. Based on meta-analysis, in AD patients, salivary levels of beta-amyloid42 and p-tau levels were significantly increased, and t-tau and lactoferrin were decreased at borderline statistical significance. However, according to pooled AUC, lactoferrin and beta-amyloid42 showed a significant predictive value for salivary-based AD diagnosis. In conclusion, potential markers such as beta-amyloid42, tau and lactoferrin can be detected in the saliva of AD patients, which could reliably support the early diagnosis of this neurodegenerative disease.

## 1. Introduction

Alzheimer’s Disease (AD) is the most common neurodegenerative disease and the leading cause of dementia [1]. Recent estimates suggest that around 50 million people suffer from dementia; however, the prognosis indicates that this number may reach 150 million by 2050 [2]. AD evolves via a progressive sequence from an asymptomatic, preclinical phase, followed by mild cognitive impairment (MCI) or mild behavioural impairment (MBI), up to AD dementia [3]. Usually, patients affected by AD develop progressive problems with episodic memory, apathy, neuropsychiatric, or mood alterations, leading to disturbances in daily living activities [4,5]. Despite several years of research, curative treatment is not available so far; therefore, the primary objective is preventing and alleviating AD risk factors [6].

The major histological hallmarks of AD include β-amyloid (Aβ) senile plaques and phosphorylated tau (p-tau) forming neurofibrillary tangles (NFTs) [7]. Furthermore, AD pathophysiology focuses on structural alterations in the synapse, synaptic damage or loss. A significant synaptic loss combined with general neuronal damage causes brain atrophy, which precedes the hallmarks mentioned above [8]. Moreover, growing evidence suggests oxidative stress, neuroinflammation, and mitochondrial dysfunction as other mechanisms of AD pathophysiology [9,10]. According to a 2018-released research framework, the diagnosis of AD should not be based on clinical symptoms but on biological biomarkers [11]. Currently, valid diagnostic tools for AD include both CSF biomarkers (Aβ_42_ and Tau) and imaging methods (MRI and PET) for the study of brain atrophy and metabolism or accumulation of pathogenic substances [12].

Saliva is an easily accessible body fluid with regular alterations in composition under different pathophysiological conditions [13]. The secretion and composition of saliva might be affected by various diseases, including gastrointestinal, thyroid, oncological, autoimmune, cardiovascular, neurological and other disorders [14,15,16,17,18,19,20,21]. Also, AD may influence these qualitative and quantitative salivary parameters. In addition, there seems to be a relationship between the human brain and saliva, which occurs via six different pathways communicating brain molecules with the saliva and vice versa. The oral–brain axis contains possible routes, such as the cranial nerves, the intranasal pathway, the lymphatic pathway, the sublingual route, the peripheral bloodstream, or the gut–brain axis with the vagus nerve [22].

Considering the beneficial aspects of saliva collection and its diagnostic values, in this systematic review, we sought to determine the quality of salivary biomarkers in AD diagnosis. In our review, we did not limit the selection of compounds based on biochemical nature but only salivary origin. Therefore, the design of this systematic review was based on the following question: “Are salivary biomarkers reliable for the diagnosis of Alzheimer’s Disease?”

## 2. Materials and Methods

### 2.1. Search Strategy and Data Extraction

Our systematic review was conducted based on the records published from 1 January 2008 to 30 September 2023, according to the Preferred Reporting Items for Systematic Reviews and Meta-Analyses (PRISMA) statement guidelines [23], using the databases PubMed, Scopus and Web of Science. The search queries included the following:

- For PubMed: saliva* AND (marker* OR biomarker* OR enzyme* OR metabolite* OR hormon*) AND (Parkinson* OR Alzheimer*);
- For Scopus: TITLE-ABS-KEY (saliva* AND (marker* OR biomarker* OR enzyme* OR metabolite* OR hormon*) AND (parkinson* OR alzheimer*));
- For Web of Science: TS = (saliva* AND (marker* OR biomarker* OR enzyme* OR metabolite* OR hormon*) AND (Parkinson* OR Alzheimer*)).

Retrieved search results were filtered by publication date after 1 January 2008. The search strategy deliberately included two major neurodegenerative diseases in connection with the planned publication of two separate papers.

Records were screened by the title, abstract and full text by two independent investigators. Studies included in this review matched all the predefined criteria according to PI(E)COS (“Population”, “Intervention”/“Exposure”, “Comparison”, “Outcomes” and “Study design”), as presented in Table 1. A detailed search flowchart is shown in the “Results” section. The study protocol was registered in the International prospective register of systematic reviews PROSPERO (CRD42023477115).

The results of the meta-analysis were presented in forest plots using the MedCalc Statistical Software, version 22.014 (MedCalc Software Ltd., Ostend, Belgium). The meta-analysis was performed for the most often biomarkers in saliva from patients with AD. The standardised mean differences and pooled AUC were calculated.

### 2.2. Quality Assessment and Critical Appraisal for the Systematic Review of Included Studies

The risk of bias in each individual study was assessed according to the “Study Quality Assessment Tool” issued by the National Heart, Lung, and Blood Institute within the National Institute of Health [24]. These questionnaires were answered by two independent investigators, and any disagreements were resolved by discussion between them.

**Figure 1:**
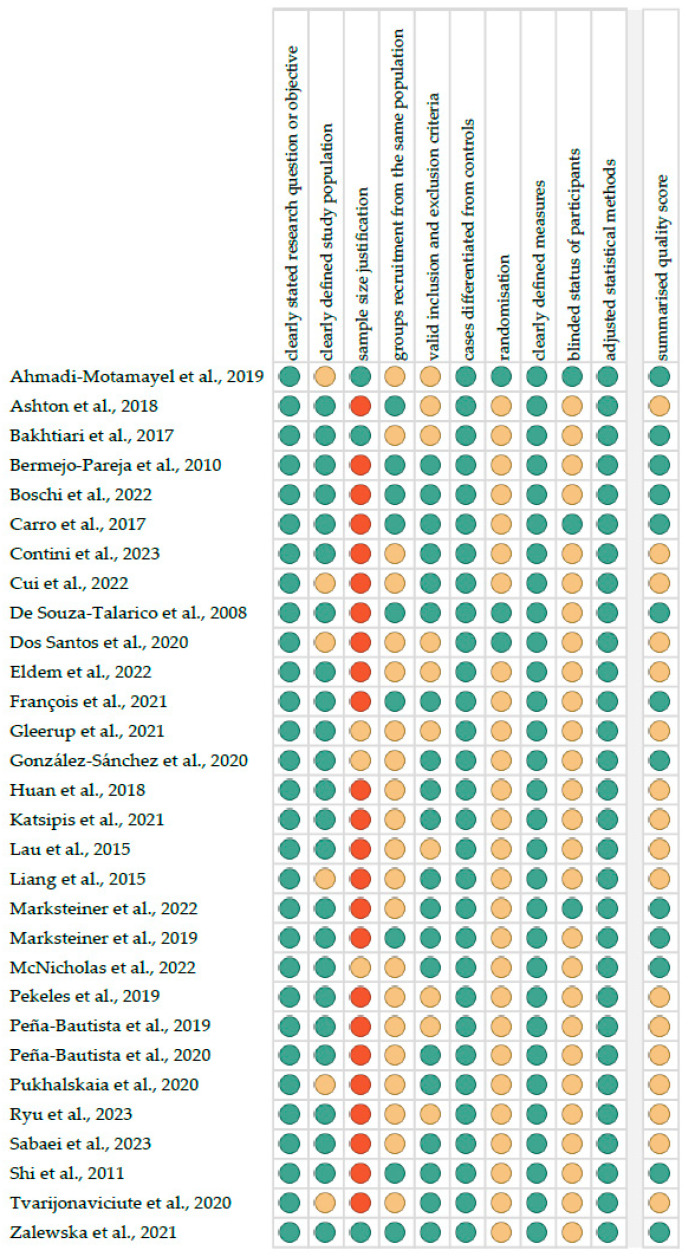
Quality assessment, including the main potential risk of bias (risk level: green—low, yellow—unspecified, red—high; quality score: green—good, yellow—intermediate, red—poor) [26,27,28,29,30,31,32,33,34,35,36,37,38,39,40,41,42,43,44,45,46,47,48,49,50,51,52,53,54,55].

Figure 1 reports the summarised quality assessment. The most frequently encountered risks of bias were the absence of data regarding sample size justification, randomisation and blinding (each for twenty-seven studies). Critical appraisal was summarised by adding up the points for each criterion of potential risk (points: 1—low, 0.5—unspecified, 0—high). Thirteen studies (43.3%) were classified as having “good” quality (≥80% total score), and seventeen (56.7%) were classified as having “intermediate” quality (≥60% total score).

All of the included studies had the third or fourth level of evidence (case-control studies), according to the five-graded scale used for classification by the Oxford Centre for Evidence-Based Medicine levels for diagnosis [25].

## 3. Results

**Figure 2:**
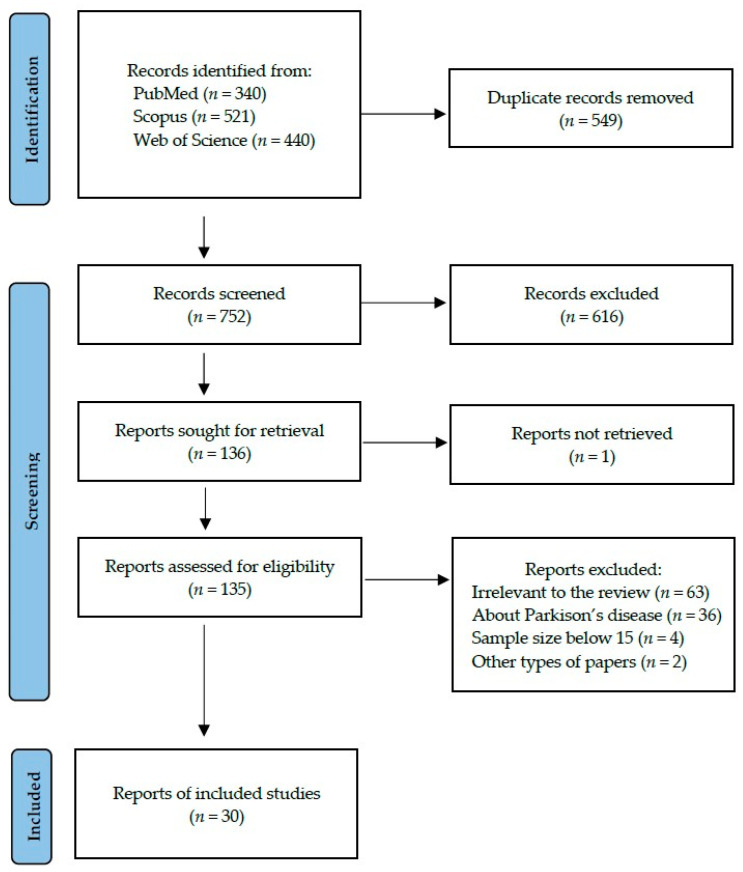
PRISMA flow diagram presenting search strategy.

Following the search criteria presented in the Section 2, our systematic review included thirty studies, demonstrating data collected in seventeen different countries from a total of 1371 participants diagnosed with Alzheimer’s Disease. Figure 2 shows the detailed selection strategy of the searched records.

In Table 2, we presented data collected from each eligible study included in the present systematic review, which included its general characteristics, such as year of publication, setting and involved participants, as well as the detailed characteristics considering types of saliva, methods of collection, centrifugation, storing and laboratory analysis, and potential salivary biomarkers for AD. Most of the studies came from Europe, which was followed by Asia. The most commonly studied material was unstimulated saliva. Very different conditions of centrifugation and storage were reported by researchers. Among the diagnostic methods, ELISA prevailed. Proteins and metabolites were the most often determined potential biomarkers. Information on inclusion and exclusion criteria of study participants and their smoking status can be found in Table S1.

Additionally, we showed the predictive parameters for most discriminant potential AD markers from the included studies in Table 3. Since not all studies reported these data and only two salivary markers were repeatable with AUC values, a meta-analysis was performed only for them. For beta-amyloid, the pooled AUC was 0.803 (SE ± 0.056), and for lactoferrin, it was 0.896 (SE ± 0.067). Both markers showed significant predictive value for salivary-based AD diagnosis (for random effects, *p*-value < 0.001).

**Figure 3:**
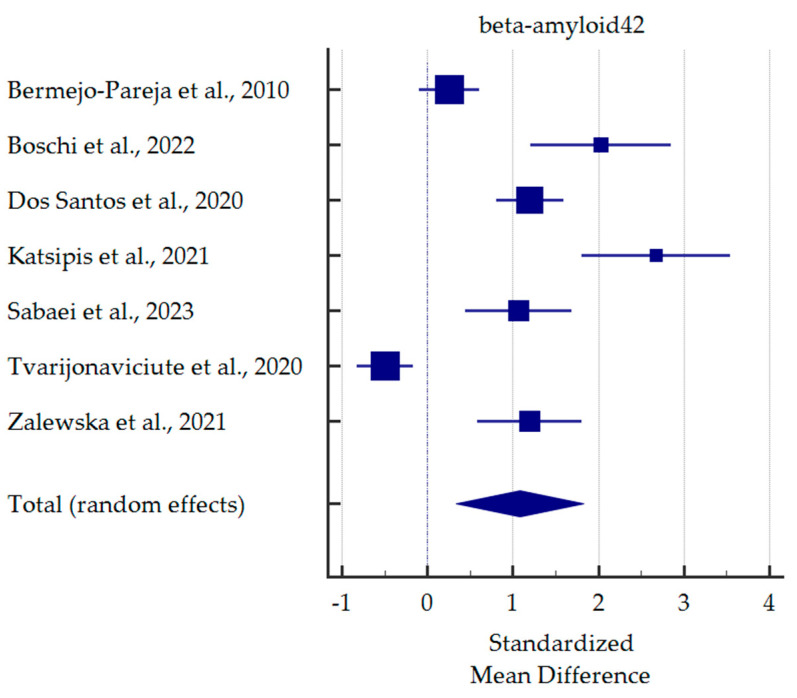
Standardised mean difference of beta-amyloid42 levels in saliva from patients with Alzheimer’s Disease compared to healthy subjects [27,28,30,31,35,37,38].

**Figure 4:**
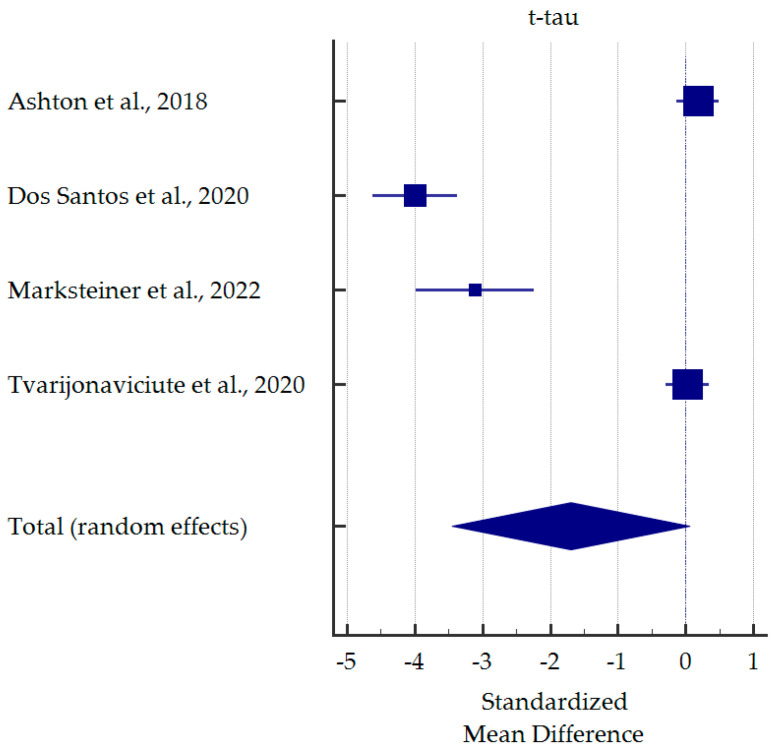
Standardised mean difference of total tau levels in saliva from patients with Alzheimer’s Disease compared to healthy subjects [26,30,33,37].

**Figure 5:**
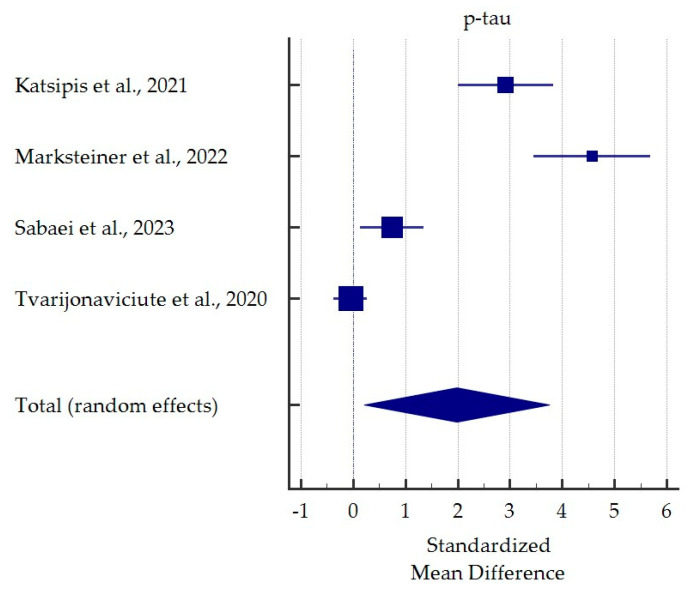
Standardised mean difference of phosphorylated tau levels in saliva from patients with Alzheimer’s Disease compared to healthy subjects [31,33,35,37].

**Figure 6:**
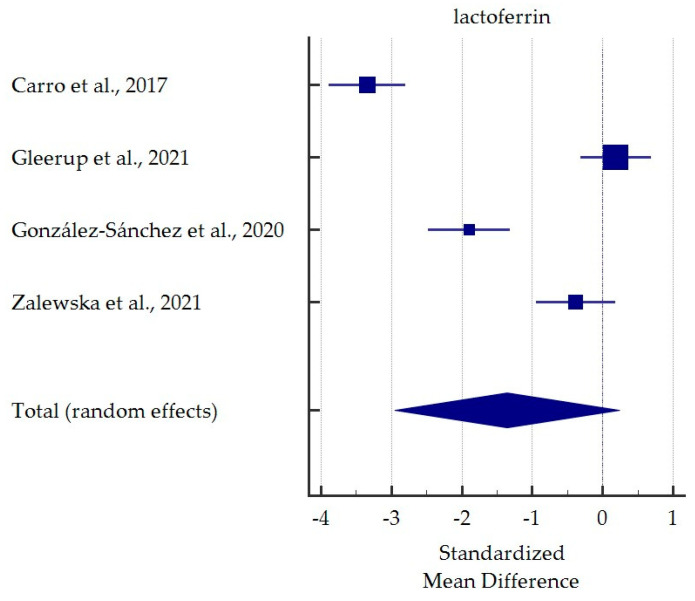
Standardised mean difference of lactoferrin levels in saliva from patients with Alzheimer’s Disease compared to healthy subjects [38,39,40,41].

A meta-analysis of differences in saliva levels between AD patients and healthy subjects was performed for the most commonly reported markers (Figure 3, Figure 4, Figure 5 and Figure 6). Both beta-amyloid42 and p-tau levels were significantly higher in the saliva of AD patients. In contrast, salivary levels of t-tau and lactoferrin were lowered in patients with AD at borderline statistical significance. Detailed standardised mean differences are presented in Table 4.

## 4. Discussion

### 4.1. β-amyloid

β-amyloid (Aβ) is a protein produced mainly in neuronal endosomes via amyloid precursor protein (APP) hydrolysis with β- and γ-secretases. In normal conditions, Aβ release is regulated by synaptic activity, which is, in turn, influenced by Aβ. Interestingly, Aβ may play an immunoprotective role [56]. Nevertheless, the accumulation of aggregated Aβ fibrils leads to the creation of Aβ plaques, which is a pathological phenomenon characteristic of AD [57]. 

In 2010, Bermejo-Pareja et al. [27] measured levels of Aβ_40_ and Aβ_42_ in the saliva of AD patients. Apart from healthy controls, 70 patients were enrolled, which were divided into three groups: the mild, moderate, and severe stages of AD (29, 24, and 17 patients, respectively). The results show that salivary levels of Aβ_42_ were significantly increased in patients in the mild AD stage. Moreover, a similar tendency was observed in moderate and severe stages although with a high standard deviation. Additionally, the authors observed a correlation between salivary Aβ_42_ concentration and sex. On the other hand, no significant differences were found in Aβ_40_ levels between AD patients and the control group.

Ten years later, another research focused on salivary Aβ_42_ levels. In this case, 60 healthy subjects and 60 patients with a probable diagnosis of AD were recruited and selected by geriatricians. There was no distinction between disease stages. Aβ_42_ levels in saliva were higher in AD patients but not significantly compared to healthy subjects [30].

In a study by Cui et al. [29], salivary Aβ_40_ and Aβ_42_ levels were assessed in a smaller sample (30 patients). Similarly, Aβ_40_ levels did not differ significantly between controls and patients, and Aβ_42_ levels were significantly increased. The performed ROC analysis revealed no significant predictive value for salivary Aβ_40_ and Aβ_42_ and their ratio. 

On the other hand, Katsipis et al. [31] measured Aβ_42_ levels in the saliva of 60 participants (20 AD patients). Again, the results indicated that salivary Aβ_42_ levels significantly increased in AD patients compared to healthy individuals and MCI patients.

Consistent results were obtained by Boschi et al. [28] in a group of 100 participants (18 AD subjects, 18 controls, 64 patients with dementia other than AD). Salivary Aβ_42_ levels were significantly elevated in patients affected by AD compared to non-demented controls. No considerable correlations between gender or MMSE score and salivary Aβ_42_ level were observed. Interestingly, salivary Aβ_42_ concentrations were significantly and negatively associated with CSF Aβ_42_ levels in all diagnostic groups except for the AD group. Nevertheless, the ROC analysis revealed satisfactory performance of salivary Aβ_42_ in AD diagnosis (AUC 0.806, specificity 68%, sensitivity 84%, with a cut-off value of 92.5 pg/mL).

Furthermore, Sabaei et al. [35] investigated salivary Aβ_1–42_ levels in the study of 70 participants, including 24 patients with mild AD. Similarly, salivary Aβ_1–42_ levels were significantly higher in AD patients in comparison with healthy controls with a slightly lower difference after age adjustment. In addition, the ROC analysis confirmed the satisfactory performance of this potential biomarker with both the cut-off point equal to 60.3 pg/mL (AUC 0.81, specificity 91%, sensitivity 62.5%) and 15.5 pg/mL (AUC 0.77, specificity 59.1%, sensitivity 91.7%).

In turn, Tvarijonaviciute et al. [37] concluded that salivary Aβ_42_ levels are decreased in AD based on the sample of 69 patients. Analysis of the univariate logistic regression models revealed that individuals with decreased Aβ_42_ levels in saliva were more likely to be in the AD group. Moreover, no significant association between disease stage and salivary Aβ_42_ level was observed. 

Another method, fluorescence of Aβ combined with the addition of Thioflavin T, was used to analyse Aβ by Zalewska et al. [38]. This research consisted of 25 controls and 25 AD patients. Concentrations of salivary Aβ were significantly higher in AD patients compared to non-demented controls (AUC 0.949, sensitivity 86.36%, specificity 84.00%).

In summary, in most studies, AD patients had elevated levels of beta-amyloid, which was statistically significant in our meta-analysis. However, in three studies, salivary Aβ_40_ and Aβ_42_ were not detected in AD patients [32,33,36]. Lau et al. [32] and Shi et al. [36] did not disclose Aβ_42_ levels employing the ELISA method and highly sensitive Luminex assays, respectively. Marksteiner et al. [33] used automated Lumipulse enzymatic light-emitting technology (Fujirebio G600II) and did not detect levels of both Aβ_40_ and Aβ_42_ in AD patients.

### 4.2. Tau

Tau belongs to the microtubule-associated protein group responsible for stabilising neuronal microtubules. In pathological conditions, tau may be hyperphosphorylated, which results in aggregation and neuronal toxicity [58]. Tau hyperphosphorylation and aggregation are connected with impaired both long- and short-term synaptic plasticity, which is a phenomenon observed in AD [59,60]. Tau protein has 85 phosphorylation sites, and in normal conditions, only 10 are phosphorylated, which is significantly less than the 55 in AD [61]. 

In 2011, Shi et al. [36] investigated both Aβ_42_ (previously mentioned) and tau levels in saliva. In comparing AD patients and controls, a non-significant decrease in t-tau concentrations in patients was observed; however, no difference was found after standardisation by total salivary protein levels. On the other hand, both absolute and standardised p-tau levels tended to increase in AD patients, but this was also insignificant. Nevertheless, a significant increase in the p-tau/t-tau ratio was observed in patients affected by AD.

Similarly, four years later, another study did not succeed in measuring salivary Aβ_42_ levels, but both p-tau and t-tau concentrations were detected. No significant differences between controls and patients with AD were found, although salivary p-tau tended to increase in the latter group. Moreover, none of these three biomarkers reflected the disease progression [32].

Interesting results were obtained by Ashton et al. [26] in a bigger sample of 53 AD patients using the Sioma platform. In contrast to the study mentioned above [36], salivary t-tau concentration tended to increase in the patients’ group compared to healthy subjects, although not significantly. In addition, the authors noticed a non-significant tendency in elevated t-tau levels associated with poorer cognitive abilities.

In a previously mentioned research study by Cui et al. [29], salivary p-tau and t-tau levels were also analysed. The Spearman rank analysis of both proteins’ salivary concentrations revealed no significant relationship. However, the p-tau/t-tau ratio increased significantly, which was consistent with a study by Shi et al. [36]. The ROC analysis showed no significant predictive value for t-tau and p-tau nor their ratio. Nevertheless, when p-tau, t-tau, Aβ_40_, and Aβ_42_ were combined, the ROC analysis revealed excellent diagnostic relevance (AUC 0.921).

On the other hand, Dos Santos et al. [30] noticed a statistically significant change in salivary t-tau levels in AD patients compared to healthy individuals. The median salivary t-tau of subjects without AD was significantly higher than that of AD patients. Conflicting results were obtained by Eldem et al. [47] in their proteomic study. In a group of 57 participants, 17 AD and 21 MCI patients were enrolled. T-tau levels were analysed using Western blot, and no significant differences between diagnostic groups were observed. Katsipis et al. [31] investigated p-tau levels in saliva. In this study, p-tau concentrations were significantly elevated in comparison not only to healthy controls but also to MCI patients.

Interestingly, although Marksteiner et al. [33] did not detect salivary Aβ_40_ and Aβ_42_ levels, the authors collected results about tau levels in saliva. T-tau levels significantly decreased in AD patients, especially in females. P-tau levels were significantly increased in MCI patients; slightly lower and not significant elevation in p-tau concentrations was observed in AD patients. Moreover, no statistically significant differences were found in the p-tau/t-tau ratio.

In 2019, Pekeles et al. [34] investigated the salivary p-tau/t-tau ratios among AD patients, MCI patients, and healthy controls, considering various phosphorylation sites. Interestingly, no significant differences were observed regarding one of the most extensively studied tau sites, T181. In contrast, analysis of both S396, S404, and a combination of S400, T403, and T404 sites showed significantly elevated levels of the p-tau/t-tau ratio in AD patients compared to the control group. S396 was most significantly increased and had better specificity than S404; however, it had worse sensitivity (S396 sensitivity 73%, specificity 50%, S404 sensitivity 83%, specificity 30%). 

In one of the most recent studies included in this review, Sabaei et al. [35] also investigated salivary p-tau concentrations. Once again, significant elevations of p-tau levels were observed in the AD group compared to healthy subjects both regardless of the age-confounding variable and after adjusting the age variable. Moreover, the ROC curve analysis revealed satisfactory performance of this biomarker (AUC 0.78, specificity 63.6%, sensitivity 91.7%).

Finally, Tvarijonaviciute et al. [37] analysed salivary p-tau and t-tau in patients suffering from AD and non-demented individuals. No significant changes were observed. P-tau tended to decrease slightly in patients compared to controls. On the other hand, t-tau reached similar values in both groups.

### 4.3. Lactoferrin

Lactoferrin (LF) is a crucial protein that plays an important role in maintaining human health [62]. Antifungal, antibacterial, antiviral, anti-carcinogenic, anti-inflammatory, and iron-binding properties enhance its relevancy in biological processes [63]. LF may have neuroprotective effects in neurodegenerative diseases, such as AD. Several mechanisms in which LF likely alleviates cognitive impairment, Aβ accumulation, and neurodegeneration were reviewed in another paper [64].

In a study by Carro et al. [39], 116 AD patients were recruited. Also, patients affected by MCI, Parkinson’s Disease (PD), and healthy controls were enrolled. Salivary LF levels were significantly lower in AD and MCI patients than in healthy controls (4.78 ± 1.11 vs. 10.24 ± 1.96 µg/mL). Moreover, a statistically significant negative correlation was found between AD and MCI severity and LF level in saliva. The analogical association was observed regarding the MMSE score. In addition, salivary LF was significantly correlated with CSF t-tau and Aβ_42_. The performed ROC analysis, which included the MCI/AD group and healthy controls, reached 100% specificity and sensitivity with a cut-off value of 7.43 µg/mL.

Consistent results were obtained by González-Sánchez et al. [41] three years later. Significantly decreased salivary LF levels were observed in MCI patients with positive amyloid-PET scans and AD patients in comparison with cognitively normal individuals. No significant differences were observed between these two experimental groups. Similarly, such differences were not found between MCI patients with negative amyloid-PET scans and controls. Additionally, no significant correlation with disease stage was noticed. Nevertheless, salivary LF performance in differentiation between AD/MCI amyloid-PET positive patients and controls, visualised via the ROC curve analysis with a cut-off value of 5.63 µg/mL, showed satisfactory results (AUC 0.952, sensitivity 86.96%, specificity 91.67%).

In a study from 2021, Zalewska et al. [38] confirmed previously mentioned results. Indeed, in a smaller sample, LF levels, measured in stimulated whole saliva and analysed in µg/mg protein unit, significantly decreased in patients suffering from AD compared to non-demented controls. In this case, AUC was 0.6896. Again, no considerable relationships were observed between LF concentrations and disease stages. Opposite findings were presented in research by Gleerup et al. [40] from the same year. In a cohort of 222 participants, 71 AD patients were included. Surprisingly, no statistically significant differences between diagnostic groups were observed. Moreover, salivary LF tended to increase in AD patients compared to healthy controls. Standardisation by the total protein concentration in saliva did not reveal considerable results. The authors suggested that the inconsistency with previous studies may have appeared due to the inclusion of more heterogeneous and milder cases, which might have contributed to LF variations in their research.

### 4.4. Acetylcholinesterase, Pseudocholinesterase, Cholinesterase

Acetylcholinesterase (AChE) is an enzyme belonging to the serine hydrolases class, which is responsible for hydrolysing acetylcholine into choline and acetic acid and, therefore, finishing the action of this neurotransmitter [65]. AChE expression is performed in several forms, including homomeric and hetero-oligomeric states. This process can be observed in various tissues: peripheral and central nervous system neurons, skeletal muscles, and endocrine or exocrine glands [66]. AChE is considered a key target for the pharmacological treatment of AD, which is focused on the inhibitors of the hydrolysis of the neurotransmitter acetylcholine [67]. Additionally, higher AChE activity has been observed in several diseases, such as lung cancer, glaucoma, ALS, Hirschsprung’s disease, pesticide poisoning, neurotoxicity, or essential hypertension [68,69,70]. 

Ahmadi-Motamayel et al. [42] investigated AChE activity in patients with AD and non-demented controls. Moreover, the authors measured the activity of pseudocholinesterase (PChE), which is a sister enzyme of AChE hydrolysing exogenous choline-based esters [42,71,72]. In a group of AD patients, salivary AChE and PChE activities were significantly elevated compared to healthy subjects. Furthermore, the increase in activity was higher in males than females, but this difference was insignificant.

Another research analysed AChE activity in the sample of 15 AD patients and 15 healthy controls. Surprisingly, AChE activity was lower in the AD group compared to controls; however, the difference was not significant. Neither age nor disease duration were clearly associated with AChE activity. Moreover, in contrast to the previous study, enzyme activity was generally lower in males than in females. It is noteworthy that all patients were on therapy with memantine, which is a neurological drug that does not inhibit AChE [43]. Discrepancies between these two studies [42,43] are difficult to explain; however, unclear methods of diagnosis establishment, memantine therapy, and differences in the number of study participants might have influenced the results. 

On the other hand, Tvarijonaviciute et al. [37] investigated salivary levels of cholinesterase. AD patients tended to have elevated levels of this enzyme compared to the control group; however, the results were not statistically significant.

### 4.5. Cortisol

Cortisol is the leading glucocorticoid hormone secreted by the adrenal cortex, fluctuating during the day [73,74]. Elevated cortisol level is associated with worse prognosis and the rapid progress of cognitive impairment in patients suffering from AD in the early stages or even the preclinical phase of the disease. Cortisol may contribute to the pathophysiology of AD by increasing both tau and Aβ pathologies as well as oxidative stress [75].

In 2008, De Souza-Talarico et al. [44] investigated salivary cortisol levels in mild AD patients (40 cases) and cognitively normal subjects (also 40 participants). Using a radioimmunoassay kit, AD patients presented significantly elevated salivary cortisol concentrations compared to controls. Slightly different times at sample collection between groups did not affect the results significantly. Interestingly, no significant correlation was observed between cortisol levels and working memory tests; however, AD patients with higher cortisol levels tended to have worse scores on one of the tests.

Different results were presented in another study published eleven years later. Peña-Bautista et al. [45] classified 97 participants into the AD group, consisting of both mild AD and MCI patients, who had positive neuroimaging and CSF biomarkers results. No significant association between AD and cortisol concentration in saliva was observed. Nevertheless, salivary cortisol levels in the AD group were increased compared to non-AD controls. 

### 4.6. Biomarkers Related to Inflammation, Oxidative Stress or Redox Imbalance 

Inflammation is clearly associated with AD pathology. Damage via various inflammatory mechanisms cumulates over years of disease progression and might considerably exacerbate pathogenic processes in this disorder [76]. Several factors participating in neuroinflammation concerning AD have been described, including cytokines, chemokines, caspases, complement system, and others [77].

Returning to research by Tvarijonaviciute et al. [37], several inflammation-related substances were also investigated. Salivary levels of haptoglobin, adenosine deaminase, and the ferric-reducing ability of plasma were decreased, whereas macrophage inflammatory protein-4, α1-antitrypsin, complement C4, and pigment epithelium-derived protein levels were increased in AD patients compared to controls. Nevertheless, only complement C4 alterations were considered significant.

On the other hand, Katsipis et al. [31] analysed salivary concentrations of glial fibrillary acidic protein (GFAP), interleukin-1β (IL-1β), IL-6, TNF-α, COX-2, and caspase-8. Interestingly, all these compounds presented significant changes between diagnostic groups. Levels of GFAP, COX-2, and caspase-8 were decreased, while IL-1β, IL-6, and TNF-α increased in patients affected by AD compared to MCI patients or healthy controls. The ROC analysis for distinguishing diagnostic groups revealed satisfactory results: between AD patients and healthy controls, AUC reached 0.998 or 1.000 (dot blot and ELISA methods, respectively), and between AD and MCI patients, AUC was 0.805 or 0.865 (dot blot and ELISA methods, respectively). Furthermore, a significant negative correlation between GFAP levels and COX-2, caspase-8, Aβ_42_, and p-tau concentrations was found. Analogically, a significant positive association was noted in regard to TNF-α, IL-1β, and IL-6 levels as well as the MMSE score.

Another study by Zalewska et al. [38] focused on several biomarkers related to inflammation, oxidative stress, or redox imbalance. Only stimulated saliva was used in this study. The ROC analysis indicated that salivary catalase, glutathione, glutathione peroxidase, the mean total antioxidant capacity/mean total oxidant status ratio (OSI), advanced glycation end products (AGEs), and IL-1β could be used to distinguish between AD patients and healthy controls clearly. The activity of salivary superoxide dismutase, glutathione peroxidase, and catalase as well as glutathione concentrations were significantly lower in the AD group compared to controls. In turn, NO, advanced oxidation protein products, AGEs, malondialdehyde, peroxynitrite, IL-1β, and nitrotyrosine concentrations, mean total oxidant status, and OSI were considerably increased in the same pattern. Moreover, a statistically significant association between the reduced activity of salivary peroxidase or superoxide dismutase and time elapsed from diagnosis of AD was observed.

On the other hand, McNicholas et al. [49] investigated the salivary levels of five inflammatory biomarkers (cystatin-C, IL-1 receptor antagonist, stratifin, haptoglobin, and matrix metalloproteinase 9) in a group of 16 AD, 15 MCI patients, and 29 non-demented controls. In general, cystatin-C, IL-1 receptor antagonist, and stratifin showed lower abundance in MCI and AD groups, whereas concentrations of haptoglobin and matrix metalloproteinase 9 were elevated. The results indicated that the levels of four of these biomarkers (without haptoglobin), adjusted for total salivary protein, were significantly altered in the AD group compared to healthy subjects, whereas only the absolute levels of haptoglobin and matrix metalloproteinase 9 were significantly changed in this comparison. Interestingly, in the MCI group, the absolute levels of all five biomarkers were significantly different compared to cognitively normal participants; however, after adjusting for total protein concentration, this significance dropped. Nevertheless, a panel consisting of the base model (only age, gender and APOEε4 allele status), cystatin-C, and IL-1 receptor antagonist (both adjusted for total protein concentration) showed excellent performance in distinguishing between AD patients and healthy controls (AUC 0.97). When matrix metalloproteinase 9 (adjusted for total protein concentration) and total protein concentration were added to this panel, it proved similar results in discriminating between either MCI or AD patients and non-demented individuals (AUC 0.97).

### 4.7. Amino Acids and Derivatives 

Amino acids play an essential role in providing communication between neurons. These compounds can contribute to neurotransmission, acting as neurotransmitters, precursors, or neuromodulators [78]. Amino acids derivatives form an interesting group with a broad correlation spectrum, including obesity or neurological diseases [78,79,80,81]. Evidence shows that patients suffering from AD have impaired neurotransmission, which might be a result of a previously described accumulation of pathological compounds [82,83]. 

Interestingly, Peña-Bautista et al. [54] measured salivary levels of several amino acids and derivatives. Participants were divided into healthy controls (12 individuals) and the AD group, which consisted of patients with MCI due to AD and mild or moderate dementia due to AD (17 and 14 participants, respectively). Salivary acetylcholine levels were significantly higher in patients with mild AD than in controls, whereas creatine and myoinositol presented significantly lower concentrations in the AD group. Moreover, salivary levels of myoinositol, acetylcholine, glutamine, and creatine were significantly correlated with neuropsychological scales. In addition, myoinositol was considerably associated with CSF Aβ level. The performed ROC analysis revealed relatively satisfactory accuracy of glutamine and acetylcholine (AUC 0.777 and 0.660, respectively). Nevertheless, a multivariate analysis with combinations of previously mentioned biomarkers indicated that a set of all these compounds (myoinositol, glutamine, creatine, acetylcholine) showed the best performance and might be used to distinguish between AD patients and healthy subjects (AUC 0.806, sensitivity 61%, specificity 92%). In this study, only glutamine presented significant differences between genders. 

In more recent research by Marksteiner et al. [33], apart from previously described tau and Aβ, norepinephrine concentrations were also investigated. The performed HPLC-EC method analysis revealed a significant decrease in salivary norepinephrine levels in AD patients compared with healthy controls.

### 4.8. miRNAs and Sirtuins

MicroRNAs (miRNAs) form a group of small endogenous non-coding RNA that regulates target gene expression [55,84]. Ryu et al. [55] investigated miRNA-485-3p concentrations in salivary exosome-enriched extracellular vesicles (EE-EV) of 27 AD patients and 13 healthy controls. The results revealed that miRNA-485-3p concentrations in salivary EE-EV from AD patients were significantly elevated compared to the control group. The ROC analysis regarding differentiating between AD and healthy individuals showed good performance of this biomarker: AUC 0.895. Moreover, statistically significant associations were observed between miRNA-485-3p concentrations in salivary EE-EV and MMSE or Aβ PET results with a stronger association with the latter ones (AUC 0.754 and 0.922, respectively).

Sirtuins (SIRT) belong to the histone deacetylases group and regulate processes like cell metabolism or gene expression via epigenetic mechanisms. Moreover, these enzymes might have neuroprotective effects [50,85]. Pukhalskaia et al. [50] enrolled 58 healthy participants and 64 AD patients in the initial or moderate stage of the disease. The results showed that SIRT1, SIRT3, and SIRT6 levels were significantly lower in the AD group compared to controls, while SIRT5 did not differ significantly. Among these biomarkers, SIRT1 and SIRT6 changed most considerably between diagnostic groups. Except for SIRT5, the rest of the mentioned SIRT significantly decreased along with patients’ age, while only SIRT1 and SIRT6 were significantly lower in older healthy subjects. 

### 4.9. Trehalose

Trehalose is a natural disaccharide which exhibits neuroprotective effects via several potential ways, including an induction of autophagy or modulation of inflammatory responses [86]. Lau et al. [32] used an improved extended gate ion-sensitive field-effect transistor (EG-ISFET) to measure salivary trehalose levels in patients suffering from AD or PD and healthy controls. The findings showed that salivary trehalose levels were higher in AD patients compared to other diagnostic groups. Furthermore, the authors stated that using the EG-ISFET method, salivary trehalose levels of the AD group could be clearly distinguished from other diagnostic groups.

### 4.10. Metabolomics and Proteomics Panel Studies

Metabolomics, which analyses and profiles metabolites in biofluids, aids in the understanding of interactions between molecules and provides insights into mechanisms underlying diseases [87,88]. Similarly, proteomics evaluates both the structures and functions of proteins to better understand their characteristics in the organism [89]. In recent years, omics research has rapidly evolved and is predicted to develop even further [90].

In 2018, Huan et al. [51] developed a salivary diagnostic model of AD based on a metabolomic approach. A total sample of 109 participants (35 cognitively healthy, 25 MCI, and 22 AD patients) was divided into two phases: discovery (to determine the most significant metabolites that differentiate paired groups) and validation (to provisionally validate selected significant metabolites detected in the discovery phase). Using top-ranked but putatively identified biomarkers, a three-element panel to distinguish between AD and healthy controls was designed and consisted of methylguanosine, choline-cytidine, and histidinyl-phenylalanine. A similar panel for discriminating between AD and MCI groups included amino-dihydroxybenzene, glucosylgalactosyl hydroxylysine—H_2_O, and aminobutyric acid + H_2_. The performed ROC analysis revealed excellent results (overall AUC 0.997, sensitivity 98.52%, specificity 96.55%, and AUC 0.993, sensitivity 100%, specificity 97.70%, respectively). Analogically, using positively identified biomarkers, the designed panels included the following: phenylalanyl-proline, phenylalanylphenylalanine, urocanic acid (AD versus controls), and alanyl-phenylalanine with phenylalanyl-proline (AD versus MCI) (AUC 0.831, sensitivity 82.22%, specificity 73.56%, and AUC 0.843, sensitivity 81.90%, specificity 72.41%, respectively).

One year later, Marksteiner et al. [53] used targeted metabolomics to study salivary metabolomic changes in AD, MCI patients, and cognitively normal individuals; each group consisted of 25 participants. The results showed decreased salivary acyl-alkyl-phosphatidyl cholines (PCae) concentrations in AD and MCI groups compared to the control group. However, only alterations in PCae C34:1-2, PCae C36:1-2-3, PCaeCC38:1–3, and PCae C40:2-3 reached significant differences when comparing AD patients and healthy subjects. It is noteworthy that the significance was especially high when all these compounds were combined. Moreover, decreased salivary levels of PCae C36:1-2-3 significantly distinguish MCI patients from controls.

Another study investigated the metabolomic and proteomic parameters of saliva collected from 80 participants (20 AD, 20 MCI patients, and 40 cognitively normal controls). Statistical analysis revealed that 79 metabolites and 346 proteins were significantly altered in a comparison between AD and control groups. Interestingly, in the MCI group, 374 proteins and only six metabolites were considered significant compared to controls. All metabolites whose levels differed significantly between the MCI/AD and control groups (L-fucose, L-tyrosine, L-ornithine, L-aspartate, rhamnose, and serotonin) were upregulated (fold change > 2.0) [48].

Interestingly, another proteomic study, described earlier in the tau section, identified transthyretin as a potential biomarker of AD. Proteomic analysis showed a significant decrease in salivary transthyretin in AD patients, which was additionally confirmed by Western blot. The results revealed a 0.5-fold reduction in both MCI and AD groups compared to the cognitively normal subjects [47]. Transthyretin is considered a highly amyloidogenic protein that is responsible for creating amyloid deposits in the nerves, heart, arterioles, or ligaments [91]. In contrast, this protein is also believed to be a neuroprotective factor in AD due to its interaction with Aβ and decrease in Aβ aggregation [92]. 

On the other hand, Liang et al. [52] performed metabolomic screening on the sample of 256 AD patients and 218 cognitively normal controls to determine salivary biomarkers of early AD. The results indicated that six biomarkers significantly differed between diagnostic groups: inosine and 3-dehydrocarnitine were downregulated, while sphinganine-1-phosphate, ornithine, hypoxanthine, and phenyllactic acid were upregulated in the AD group compared with controls. Furthermore, the ROC analysis revealed that sphinganine-1-phosphate, ornithine, and phenyllactic acid seem most promising as salivary biomarkers of AD (AUC 0.998, sensitivity 99.4%, specificity 98.2%; AUC, 0.927 sensitivity 81.9%, specificity 90.7%; AUC 0.831, sensitivity 79.5%, specificity 84.3%, respectively); whereas inosine, 3-dehydrocarnitine, and hypoxanthine proved worse performance (AUC 0.740, sensitivity 66.8%, specificity 77.0%; AUC 0.669, sensitivity 57.4%, specificity 84.2%; AUC 0.674, sensitivity 53.7%, specificity 73.9%, respectively).

In a recent study, Contini et al. [46] enrolled 36 patients affected by PD, 36 healthy controls, and 35 AD patients (13 in moderate and 22 in mild disease stage). Using a proteomic approach, significant differences between various compounds in diagnostic groups were observed. AD patients had significantly higher abundances of thymosin β4, α-defensins—1, 2, 3, and sum of α-defensins, histatin 1 mono- and non-phosphorylated, statherin 2P, des 1-9 and des 1-13, P-C peptide, cystatin A, B-SSG, total cystatin B monomer, cystatin B S-S dimer, total cystatin B, S100A8-SNO, sum of S100A8-A8SNO, S100A9s, sum of S100A9s, and total S100A9 (s + l) compared to controls, and there were analogically significantly lower abundances of PRP1 0P. Moreover, 24 biomarkers were determined to distinguish between patients suffering from AD and PD—respectively, higher expression of α-defensins—1, 2, and sum, Hst1, Hst1 0P, Hst5, Hst6, statherin 2P, 1P, desD1, des1-9, des1-10 and des1-13, PRP1 1P, PRP3 2P, PRP3 1P, P-C peptide, cystatin SN and S100A9sox, and lower expression of SLPI, PB des1-5 and des1-12, SV1 and cystatin SA.

### 4.11. PD-Related Biomarkers in AD 

One of the primary hallmarks of PD is α-synuclein [93]. Interestingly, in a previously mentioned study, Sabaei et al. [35] observed significantly decreased salivary total α-synuclein levels in AD patients compared to healthy controls either without age-confounding variables or after adjusting for age. Nevertheless, the ROC analysis with a cut-off point equal to 9.4 pg/mL did not prove the high reliability of this biomarker in AD diagnosis (AUC 0.71, sensitivity 66.7%, specificity 68.2%). 

On the other hand, heme oxygenase-1 (HO-1) is associated with both AD and PD, since HO-1 dysregulation is linked with neuroinflammation presented in both disorders [94]. In a study by Galindez et al. [95], patients suffering from both diseases were included along with patients affected by other neurological disorders and healthy controls. Importantly, AD patients were combined together with MCI patients in one group. The results indicated that this group had significantly higher salivary HO-1 levels than healthy controls. After combining AD, MCI, and PD patients in one group (neurodegenerative) and non-neurodegenerative individuals in another, the ROC analysis revealed satisfactory results in distinguishing between neurodegenerative and non-neurodegenerative subjects (AUC 0.86, sensitivity 79%, specificity 80%).

### 4.12. Study Limitations

The limitations of the study include the heterogeneity of the included studies in terms of diagnostic methods and inclusion and exclusion criteria for participants (e.g., demographic characteristics, diagnosis criteria). Only some researchers conducted and reported the results of ROC analysis to assess the predictive reliability of potential salivary markers. Moreover, the diversity of the biomarkers studied made it difficult to compare their usefulness. In general, we meta-analysed repeated markers, but the others that appeared in individual studies were also discussed.

## 5. Conclusions

In conclusion, some potential biomarkers such as beta-amyloid42, t-tau, p-tau and lactoferrin could be detected in the saliva of patients with Alzheimer’s Disease. Therefore, these protein molecules could reliably support the early diagnosis of neurodegenerative diseases. However, further research is necessary to confirm these findings and to search for the predictive ability of other substances.

## Figures and Tables

**Table 1:** Inclusion and exclusion criteria according to the PI(E)COS.

Parameter	Inclusion Criteria	Exclusion Criteria
Population	Patients aged 0–99 years, both genders; Sample size: 15 patients or more	Sample size: below 15 patients or controls
Intervention/Exposure	Alzheimer’s Disease	Other diseases, e.g., MCI
Comparison	Non-demented control group	Lack of non-demented control group
Outcomes	Alterations in salivary markers level	Alterations in other markers level (e.g., serum), microbiota
Study Design	Case control, cohort, and cross-sectional studies	Literature reviews, case reports, expert opinion, letters to the editor, conference reports
	Published after 1 January 2008	Not published in English

**Table 2:** The characteristics of included studies.

Author, Year	Setting	Study Group (F/M), Age	Control Group (F/M), Age	Type of Saliva and Method of Collection	Centrifugation and Storing	Method of Marker Determination	Salivary Biomarkers
Ashton et al., 2018 [26]	UK	AD: 53 (30/23), 81.4 ± 6.6; MCI: 68 (35/33), 79.8 ± 6.4	160 (94/66), 78.0 ± 6.7	unstimulated saliva collected into preweighed sterile plastic 30 mL containers at 30 s intervals for 2 min or until 2 mL achieved, overnight fasting	centrifuged at 500× *g* for 10 min at 4 °C, immediately stored at −80 °C	Human Total Tau assay on an HD-1 Simoa instrument	t-tau (ns)
Bermejo-Pareja et al., 2010 [27]	Spain	AD: 70 (49/21), 77.20 (60–91); PD: 51 (25/26); 72.96 (60–93)	56 (39/17), 74.35 (64–85)	approx. 1 mL of saliva collected at least 4 h after eating or drinking (at around 1 a.m.) in sterile plastic containers previously treated with 2% sodium azide solution	centrifuged at 1500 rpm for 5 min, immediately frozen at −80 °C until used	ELISA	Aβ42, Aβ40 (ns)
Boschi et al., 2022 [28]	Italy	AD: 18 (10/8), 72.13 ± 5.45	18 (11/7), 65.67 ± 12.02	10 mL of whole, unstimulated saliva collected in a 15 mL polypropylene Falcon tube, all patients fasting for at least 8 h	immediately placed on ice, precleared by a low spin at 600× *g* for 10 min at 4 °C, stored at −80 °C	ELISA	Aβ42
Cui et al., 2022 [29]	China	AD: 30 (NR), NR	30 (NR), NR	unstimulated/stimulated whole/sublingual/submandibular/parotid saliva collected in pre-chilled polypropylene tubes on ice between 9 and 9:30 a.m. using a Salivette, 5 mL in total	centrifuged at −20 °C, transferred to the laboratory on regular basis	ELISA	Aβ42, Aβ40 (ns), p-tau (ns), t-tau (ns)
Dos Santos et al., 2020 [30]	Brazil	AD: 60 (NR), NR	60 (NR), NR	collected using Salivette, placed for 3 min in the mouth without chewing	stored in a cooler at −20 °C, centrifuged at 3000 rpm for 15 min	ELISA	Aβ42, t-tau
Katsipis et al., 2021 [31]	Greece	AD: 20 (9/11), 75 ± 5.5; MCI: 20 (12/8), 75 ± 7.2	20 (11/9), 79 ± 4.7	unstimulated saliva collected in the morning, by passive drooling	centrifuged at 13,500 rpm for 15 min, stored at −80 °C	ELISA, Dot Blot, Western Blot	GFAP, p-tau, IL-1β, IL-6, TNF-α, caspase-8, COX-2, Aβ42
Lau et al., 2015 [32]	Korea	AD: 20 (12/8), 72.5 ± 7.68; PD: 20 (11/9), 73 ± 8.07	20 (15/5), 66.1 ± 7.79	3 mL unstimulated saliva collected after at least 4 h of fasting	centrifuged at 1000× *g* for 15 min, stored at −80 °C	ELISA, EG-ISFET	Aβ42 (not detected), p-tau (ns), t-tau (ns), trehalose (undefined)
Marksteiner et al., 2022 [33]	Austria	AD: 44 (25/19), 79 ± 1; MCI: 45 (25/20), 74 ± 1	27 (14/13), 71 ± 1	approx. 1.5 mL of saliva collected in the early morning using Salivette kept 2 min in the mouth	centrifuged at 3000× *g* 5 min, stored at −80 °C	HPLC-EC, robotic-automated enzymatic Lumipulse assay	norepinephrine, p-tau (ns), t-tau, Aβ40 and Aβ42 (not detected in cases)
Pekeles et al., 2019 [34]	Canada	AD: 46 (22/24), 80 (9); MCI: 55 (32/23), 78 (14)	47 (32/15), 73 (6)	4–5 mL of unstimulated saliva collected in the morning by spitting into a sterile 50 mL polypropylene tube	put on ice, then in 100 °C water bath for 20 min, centrifuged at 5000 rpm or 10,000 rpm for 10 min at 4 °C, stored at −80 °C	Western Blot, tau antibodies	p-tau (T181 (ns), S396, S404, S400, T403, T404)
Sabaei et al., 2023 [35]	Iran	AD: 24 (10/14), 73.5 ± 9.8; PD: 24 (10/14), 61.2 ± 8.7	22 (13/9), 64.1 ± 9.2	dental cotton roll placed on the oral side of the cheek to, moist rolls located inside the salivary collector tubes	centrifuged at 1500 rpm for 5 min, stored at −80 °C	ELISA	Aβ 1–42, p-tau, α-synuclein
Shi et al., 2011 [36]	USA	AD: 21 (11/10), 68.8 (52–85)	38 (19/19), 69.0 (40–88)	collected by placing a dental cotton roll between the cheek and gum of the mouth for at least 1 min, then spun inside a Salivette	stored at −70 °C	Luminex assays, IP/MS	t-tau, p-tau, Aβ42 (not detected)
Tvarijonaviciute et al., 2020 [37]	Spain	AD: 69 (NR), NR	83 (NR), NR	up to 0.5 mL of unstimulated whole saliva collected passively by drooling into a propylene tube, between 9 and 12 a.m.	centrifuged at 3000× *g* for 10 min at 4 °C, stored at −80 °C	MILLIPLEX MAP, automated spectrophotometric method	FRAP (ns), ADA (ns), ChE (ns), Hp (ns), Aβ42, Aβ40 (ns), t-tau (ns), p-tau (ns), CRP (ns), PEDF (ns), SAP (ns), MIP-4 (ns), CC4, α1 antitrypsin (ns)
Zalewska et al., 2021 [38]	Poland	AD: 25 (15/10), 81.19 ± 6.77	25 (15/10), 82.1 ± 6.67	stimulated whole saliva collected after drinking a glass of water and a 5 min conversation, saliva taken with a pipette after spraying of 100 μL of citric acid on the tip of the tongue every 30 s for 10 min	placed on ice, centrifuged at 5000× *g* for 20 min at 4 °C within 30 min from collection, stored at −84 °C	colorimetric, spectrofluorimetric, spectrophotometric methods, thioflavin T fluorescence, ELISA	Aβ, lactoferrin, IL-1β, SOD, CAT, GPx, UA (ns), GSH, TAC (ns), TOS, AGE, AOPP, MDA, NO, peroxynitrite, nitrotyrosine
Carro et al., 2017 [39]	Spain	AD: 80 (49/31), 76.2 ± 5.33; MCI: 44 (25/19), 75.16 ± 5.13; PD: 59 (32/27), 69.5 ± 8.6	91 (59/32), 73.7 ± 6.88	0.5 mL of unstimulated whole saliva collected into sterile plastic containers precoated with 2% sodium azide solution	immediately placed on ice, precleared by a low spin at 600× *g* for 10 min at 4 °C, stored at −80 °C	ELISA	lactoferrin
Gleerup et al., 2021 [40]	Denmark	AD: 71 (41/30), 72.1 ± 7.3; MCI: 56 (27/29), 70.4 ± 8.2	20 (8/12), 65.7 ± 10.1	1–3 mL of unstimulated whole saliva, collected between 9:15 and 10:15 a.m., or around noon, in a 15 mL polypropylene falcon tube	placed on ice, centrifuged at 2000 rpm for 10 min at 4 °C, stored at −80 °C	ELISA	lactoferrin (ns)
González-Sánchez et al., 2020 [41]	Spain	AD-PET+: 25 (12/13), 67.2 ± 9.2, MCI-PET+: 21 (8/13), 68.8 ± 7.5	Control-PET-: 48 (33/15), 66.9 ± 5.9; control-PET+: 4 (2/2), 75.9 ± 3.6	0.5 mL of unstimulated whole saliva collected into sterile plastic containers precoated with 2% sodium azide solution	immediately placed on ice, precleared by a low spin at 600× *g* for 10 min at 4 °C, stored at −80 °C	ELISA	lactoferrin
Ahmadi-Motamayel et al., 2019 [42]	Iran	AD: 30 (NR), 56–85	30 (NR), 57–85	unstimulated, whole saliva collected in falcon tubes within 5–10 min, from 8 to 10 a.m.	centrifuged at 3000 rpm for 10 min, stored at −80 °C	spectrophotometric assay using the Ellman colorimetric method	AChE, PChE
Bakhtiari et al., 2017 [43]	Iran	AD: 15 (6/9), 78.4 (64–90)	15 (8/7), 71 (61–85)	2 mL of whole, unstimulated saliva samples collected by spitting into 15 mL Falcon tubes, between 9 and 12 a.m.	immediately placed on ice and stored at −70 °C, centrifuged at 3000 rpm for 10 min	Ellman colorimetric method	AChE (ns)
De Souza-Talarico et al., 2008 [44]	Brazil	AD: 40 (27/13), 80.1 ± 6.0	40 (35/5), 72.2 ± 6.3	drawn off with pipette and transferred to a sterile tube, collected within 2 h of waking in the next day after cognitive evaluation	centrifuged at 2200 rpm for 15 min at 4 °C, immediately placed on chipped ice, stored at −20 °C	radioimmunoassay	cortisol
Peña-Bautista et al., 2019 [45]	Spain	mild AD: 50 (29/21), 70 (68, 74); MCI-AD: 47 (30/17), 71 (69, 74)	41 (16/25), 66 (62, 69)	according to previously described procedures, collected between 8 and 10 a.m.	stored at −80 °C, thawed on ice, homogenized and centrifuged at 3500× *g* for 10 min at 4 °C	UPLC-MS/MS	cortisol (ns)
Contini et al., 2023 [46]	Italy	AD: 35 (23/12), 80 ± 6; PD: 36 (11/15), 72 ± 7	36 (18/18), 78 ± 6	unstimulated whole saliva collected between 9 and 12 a.m., with a soft plastic aspirator for less than 1 min, transferred to a plastic tube cooled on ice	centrifuged 20,000× *g* for 15 min at 4 °C, stored at −80 °C or immediately analysed	RP-HPLC-LR-ESI-MS	proteomics
Eldem et al., 2022 [47]	Switzerland	AD: 17 (9/8), 72 ± 1.36; MCI: 21 (16/5), 73 ± 1.51	19 (13/6), 64 ± 2.63	2 mL of whole unstimulated saliva collected between 9 and 11 a.m.	stored at –20 °C	LC-MS/MS, Western Blot, FASP	proteomics, t-tau (ns), transthyretin
François et al., 2021 [48]	Australia	AD: 20 (8/12), 78.0; MCI: 20 (11/9), 77.8	40 (19/21), 75.3	collected using RNAProSAL	stored at −80 °C	GC-MS, LC-MS	proteomics, metabolomics
McNicholas et al., 2022 [49]	Australia	AD: 16 (6/10), 79 ± 6; MCI: 15 (8/7), 76 ± 6	29 (14/15), 74 ± 8	collected using RNAProSAL	stored at −80 °C	ELISA	cystatin-C, IL-1 receptor antagonist, stratifin, haptoglobin, matrix metalloproteinase 9
Pukhalskaia et al., 2020 [50]	Russia	AD: 64 (NR), elderly 63.0 ± 2.4, senile 82.0 ± 2.3	58 (NR), NR	collected between 10 and 12 a.m.	NR	ELISA	SIRT1, SIRT3, SIRT5 (ns), SIRT6
Huan et al., 2018 [51]	Canada	AD: 22 (16/6), 77.09 ± 11.20; MCI: 25 (15/10), 70.40 ± 3.38	35 (22/13), 69.94 ± 3.80; 10 (5/5), 71.40 ± 2.84	whole saliva collected using Oragene DNA Self-Collection Kit OG-500, sample placed inside the kit, shaken	stored at −80 °C	LC-MS	metabolomics
Liang et al., 2015 [52]	China	AD: 256 (NR), NR	218 (NR), NR	collected between 9 and 11 a.m.	centrifuged at 10,000 rpm for 20 min at 4 °C, stored at −80 °C	FUPLC-MS	metabolomics
Marksteiner et al., 2019 [53]	Austria	AD: 25 (17/8), 80.4 ± 7.2; MCI: 25 (16/9), 75.9 ± 8.8	25 (16/9), 74.8 ± 4.4	1–2 mL of saliva collected in the early morning by spitting into a 15 mL sterile falcon tube for 2 min	stored at −80 °C until analysis, centrifuged at 14,000× *g* for 5 min	FIA-MS/MS, using the AbsoluteIDQ p150 Kit	metabolomics
Peña-Bautista et al., 2020 [54]	Spain	mild AD: 14 (NR), NR; MCI-AD: 17 (NR), NR	12 (NR), NR	1–2 mL of whole-mouth saliva collected by spitting into sterile bottles between 10 and 12 a.m.	stored at −80 °C, centrifuged at 1200× *g* for 5 min at 4 °C	UPLC-MS/MS	aspartic acid (ns), glutamic acid (ns), glutamine, GABA (ns), creatine, taurine, N-acetyl aspartate (ns), myoinositol, acetylcholine
Ryu et al., 2023 [55]	South Korea	AD: 27 (12/15), 72.59 ± 6.90	13 (11/2), 75.46 ± 6.58	collected by oral swabs, transferred to sterilised tubes	centrifuged at 12,000× *g* for 10 min at 4 °C, stored at −70 °C	qPCR	miRNA-485-3p

Legend: Aβ, beta-amyloid; AD, Alzheimer’s Disease; ADA, adenosine deaminase; AGE, advanced glycation end products; AOPP, advanced oxidation protein products; CC4, complement C4; CAT, catalase; ChE, cholinesterase; COX-2, cyclooxygenase 2; CRP, C-reactive protein; ELISA, enzyme-linked immunosorbent assay; FRAP, ferric-reducing ability of plasma; GABA, gamma-aminobutyric acid; GFAP, glial fibrillar acidic protein; GSH, glutathione; GPx, glutathione peroxidase; Hp, haptoglobin; IL, interleukin; IP/MS, immunoprecipitation/mass spectrometry identification; LC-MS/MS, liquid chromatography/mass spectroscopy; LF, lactoferrin; MCI, mild cognitive impairment; MDA, malondialdehyde; MIP-4, macrophage inflammatory protein-4; NO, nitric oxide; NR, not reported; ns, not significant; OSI, oxidative stress index; PChE, pseudocholinesterase; PEDF, pigment epithelium-derived protein; PGs, prostaglandins; p-tau, phosphorylated tau; qPCR, quantitative real-time polymerase chain reaction; SAP, salivary amyloid A; SIRT, sirtuin; SOD, superoxide dismutase; TAC, mean total antioxidant capacity; t-tau, total tau; TNF-α, tumour necrosis factor-alpha; TOS, total oxidant status; UA, uric acid; UPLC-MS/MS, ultra-performance liquid chromatography coupled to tandem mass spectrometry; UK, the United Kingdom; USA, the United States of America.

**Table 3:** Reported predictive parameters of most discriminant potential biomarkers for Alzheimer’s Disease (vs. healthy subjects) from included studies.

Study	Most Discriminant Markers	AUC	−95% CI	+95% CI	Sensitivity (%)	Specificity (%)
Boschi et al., 2022 [28]	Aβ42	0.806	-	-	84	68
Carro et al., 2017 [39]	lactoferrin	1	1	1	100	100
Cui et al., 2022 [29]	Aβ42	0.8483	-	-	-	-
	p-tau, t-tau, Aβ40, Aβ42 combined together	0.9211	-	-	-	-
González-Sánchez et al., 2020 [41]	lactoferrin	0.93	0.876	0.989	87.1	92.9
Huan et al., 2018 [51]	methylguanosine, choline-cytidine, histidinyl-phenylalanine	0.997	0.997	1.000	98.5	96.6
	phenylalanyl-proline, phenylalanylphenylalanine, urocanic acid	0.831	0.770	0.888	82.2	73.6
Katsipis et al., 2021 [31]	GFAP (ELISA)	1.000	1.000	1.000	75	100
	GFAP (Dot Blot)	1.000	1.000	1.000	85	75
Liang et al., 2015 [52]	sphinganine-1-phosphate	0.998	-	-	99.4	98.2
	ornithine	0.927	-	-	81.9	90.7
	phenyllactic acid	0.831	-	-	79.5	84.3
	inosine	0.740	-	-	66.8	77.0
	3-dehydrocarnitine	0.669	-	-	57.4	84.2
	hypoxanthine	0.674	-	-	53.7	73.9
Peña-Bautista et al., 2020 [54]	acetylcholine	0.660	0.492	0.828	-	-
	glutamine	0.777	0.619	0.935	-	-
	creatine	0.331	0.167	0.494	-	-
	myoinositol	0.261	0.113	0.408	-	-
	myoinositol, glutamine, creatine, acetylcholine combined together	0.806	0.674	0.939	61	92
Ryu et al., 2023 [55]	miRNA-485-3p	0.895	0.796	0.994	74.1	92.3
Sabaei et al., 2023 [35]	Aβ1–42	0.81	-	-	62.5	91.0
	p-tau	0.78	-	-	91.7	63.6
	α-synuclein	0.71	-	-	66.7	68.2
Zalewska et al., 2021 [38]	CAT	0.918	0.827	1.000	82.6	84.0
	GPx	0.741	0.584	0.897	73.9	72.0
	GSH	0.684	0.526	0.842	72.7	72.0
	IL-1β	0.853	0.742	0.964	84.0	84.0
	Aβ	0.949	0.894	1.000	86.4	84.0
	lactoferrin	0.690	0.537	0.842	64.0	64.0
	SOD	0.777	0.629	0.926	69.6	68.0
	TOS	0.920	0.814	1.000	91.3	92.0
	AGE	0.936	0.851	1.000	87.0	88.0
	AOPP	0.680	0.531	0.829	56.0	56.0
	MDA	0.688	0.508	0.868	66.7	68.0
	NO	0.672	0.523	0.821	56.0	56.0
	nitrotyrosine	0.702	0.541	0.863	63.6	64.0
	peroxynitrite	0.816	0.693	0.940	63.6	79.2
	OSI	0.936	0.847	1.000	90.0	92.0

Legend: Aβ, beta-amyloid; AGE, advanced glycation end products; AOPP, advanced oxidation protein products; AUC, area under curve; CAT, catalase; CI, confidence interval; ELISA, enzyme-linked immunosorbent assay; GFAP, glial fibrillar acidic protein; GPx, glutathione peroxidase; GSH, glutathione; IL-1β, interleukin 1 beta; MDA, malondialdehyde; NO, nitric oxide; OSI, oxidative stress index; p-tau, phosphorylated tau; SOD, superoxide dismutase; TOS, mean total oxidant status; t-tau, total tau.

**Table 4:** Detailed results for meta-analysis comparing salivary levels of the most often potential markers for Alzheimer’s Disease vs. healthy subjects.

Study	SMD	95% CI	*p*-Value	Weight
Beta-amyloid42				
Bermejo-Pareja et al., 2010 [27]	0.254	−0.100 to 0.609		15.11
Boschi et al., 2022 [28]	2.025	1.204 to 2.846		13.29
Dos Santos et al., 2020 [30]	1.198	0.807 to 1.588		15.01
Katsipis et al., 2021 [31]	2.676	1.804 to 3.548		13.02
Sabaei et al., 2023 [35]	1.064	0.438 to 1.690		14.16
Tvarijonaviciute et al., 2020 [37]	−0.497	−0.822 to −0.172		15.19
Zalewska et al., 2021 [38]	1.192	0.584 to 1.801		14.22
Total (random effects)	1.080	0.334 to 1.827	0.005	
t-tau				
Ashton et al., 2018 [26]	0.179	−0.132 to 0.491		25.47
Dos Santos et al., 2020 [30]	−4.000	−4.625 to −3.375		24.88
Marksteiner et al., 2022 [33]	−3.110	−3.986 to −2.233		24.20
Tvarijonaviciute et al., 2020 [37]	0.022	−0.299 to 0.342		25.46
Total (random effects)	−1.696	−3.453 to 0.060	0.058	
p-tau				
Katsipis et al., 2021 [31]	2.916	2.005 to 3.827		24.63
Marksteiner et al., 2022 [33]	4.569	3.445 to 5.693		23.85
Sabaei et al., 2023 [35]	0.741	0.136 to 1.346		25.50
Tvarijonaviciute et al., 2020 [37]	−0.053	−0.374 to 0.267		26.02
Total (random effects)	1.983	0.209 to 3.757	0.029	
Lactoferrin				
Carro et al., 2017 [39]	−3.346	−3.889 to −2.803		25.00
Gleerup et al., 2021 [40]	0.189	−0.310 to 0.689		25.12
González-Sánchez et al., 2020 [41]	−1.897	−2.475 to −1.318		24.92
Zalewska et al., 2021 [38]	−0.382	−0.947 to 0.183		24.97
Total (random effects)	−1.357	−2.953 to 0.239	0.095	

## Data Availability

Data are available on request from the corresponding author.

## Supplementary Materials

The following supporting information can be downloaded at https://www.mdpi.com/article/10.3390/ijms25021168/s1.
