# Supplementary material for: Salivary Biomarkers for Alzheimer’s Disease: A Systematic Review with Meta-Analysis
Source: Int J Mol Sci. 2024 Jan 18;25(2):1168. doi: 10.3390/ijms25021168 (PMC10817083; doi:10.3390/ijms25021168)
Supplement: Supplementary file 1 [file ijms-25-01168-s001.zip › ijms-2786686-supplementary.pdf]

Table S1. The detailed characteristics of included studies.

Study	Inclusion Criteria	Exclusion Criteria	Smoking Status
Ahmadi-Motamayel et al., 2019	AD diagnosis, edentulous	any other systemic disease, periodontal disease, medications other than neurologic drugs, other dementias, smoking, poor oral hygiene; HC: any cognitive symptoms, any sign or symptom of dementia	non-smokers
Ashton et al., 2018	AD: diagnosis of probable AD according to Diagnostic and Statistical Manual for Mental Diagnosis	NR	NR
Bakhtiari et al., 2017	AD: AD diagnosis, on memantine therapy	HC: neurological or cognitive disease	NR
Bermejo-Pareja et al., 2010	AD: AD diagnosis according to the DSM-IV or NINCDS-ADRDA criteria; HC: completely normal cognitive and functional level; PD: PD diagnosis according to appropriate criteria	AD: vascular dementia	NR
Boschi et al., 2022	AD: AD diagnosis based on the NIA-AA criteria, A-T-N classification scheme; non-AD: normal biomarker profile, evidence of non-Alzheimer's pathologic change	HC: dementia or cognitive impairment	NR
Carro et al., 2017	HC: CDR scale = 0, MMSE score = 29 (0.8), normal yields in cognitive tests of memory, language, executive functions, AD: AD diagnosis according to the NINCDS-ADRDA criteria; MCI: MCI diagnosis based on the Petersen criteria	HC: psychiatric, systemic or neurological disorders that can affect cognition or limit participation in the study, visual, auditory, language limitations	NR
Contini et al., 2023	AD: AD diagnosis according to the NIA-AA criteria	major oral disease (periodontitis, caries, dry mouth), history of radiotherapy or chemotherapy, antidepressants or anticholinergic drugs	NR
Cui et al., 2022	AD: AD diagnosis according to the CCMD-3 and Diagnosis and Statistics Manual of Mental Illness criteria; HC: normal cognitive function; age more than or equal to 60 years in both groups	AD: severe organ dysfunction, having taken antipsychotic drugs a month before; HC: mental disorder, hereditary neurological disease among immediate family members, history of head trauma	NR
De Souza-Talarico et al., 2008	AD: fulfillment of the NINCDS-ADRDA criteria for probable AD, mild dementia according to the DSM-III-r criteria	HC: other neurological or psychiatric disease, cognitive alterations incompatible with the norm for age, history of alcohol or drug abuse, using psychoactive medication, illiterate individuals	NR
Dos Santos et al., 2020	AD: probable AD diagnosis, all stages of disease	signs of mouth injury at the time of saliva collection	NR

Eldem et al., 2022	AD, MCI: diagnosis based on recommendations of Swiss memory clinics for the diagnosis of dementia; categorization of participants according to CDR, MMSE, UPSIT scales	NR	NR
François et al., 2021	AD: AD diagnosis according to the NINCDS-ADRDA, NIA-AA criteria; MCI: MCI diagnosis according to the NINCDS-ADRDA, NIA-AA criteria	significant cognitive comorbidities including head trauma, learning disability, alcoholism, PD, chemotherapy/radiotherapy treatment for cancer, taking micronutrient supplements (folate, vitamin B12) above recommended intakes	NR
Gleerup et al., 2021	AD: AD diagnosis according to the NIA-AA criteria; MCI: MCI diagnosis according to the International Working Group in Mild Cognitive Impairment criteria, non-AD patients diagnosed according to appropriate criteria, HC: no fullfillment of above mentioned criteria	NR	NR
González-Sánchez et al., 2020	controls: age over 60, MMSE score over 27, no history or clinical signs of neurological or psychiatric disease, preservation of social and functional abilities; patients: diagnosis according to appropriate criteria	concomitant psychiatric, neurological or non-neurological medical conditions, medications that could affect cognition	NR
Huan et al., 2018	MCI and HC: two waves (4.5 years) of longitudinal data, complete data on the Victoria Longitudinal Study cognitive status reference battery, minimum MMSE = 24; AD: AD diagnosis based on the DSM-IV criteria	MCI and HC: diagnosed dementia, cardiovascular disease, stroke history, or psychiatric illness; AD: vascular dementia	NR
Katsipis et al., 2021	AD: AD diagnosis according to the NIA-AA criteria; MCI: MCI diagnosis based on the Petersen criteria, HC: no neurological disease	abnormal levels of vitamin B9 or B12, and thyroid-stimulating hormone (TSH), dementia due to syphilis, stroke tumor, autoimmune disorders, chronic inflammatory diseases, active periodontal lesions, periodontitis	NR
Lau et al., 2015	HC: at least 50 years of ager, good general health, no history of neurological, psychiatric or major medical diagnosis that could contribute significantly to dementia or cognitive impairment; series of clinical and neuropsychological examinations by physicians and neuropsychologists defined	NR	AD: 3 smokers, 1 ex-smoker; HC: 3 smokers, 1 ex-smoker

	participants' inclusion to appropriate groups		
Liang et al., 2015	AD: AD diagnosis based on the NINCDS-ADRDA criteria	HC: neurologic or cognitive disease	NR
Marksteiner et al., 2022	AD: AD diagnosis based on the NINCDS-ADRDA criteria; MCI: MCI diagnosis according to the Petersen criteria; patients' diagnosis according to the previous work	another primary neurological or mental disorder, any kind of metabolic decompensation, any sign of peripheral inflammation, longterm alcohol, drug abuse, any current, clinically significant cardiovascular disease, current intake of medication that could impact saliva production or saliva composition	NR
Marksteiner et al., 2019	AD: AD diagnosis; MCI: MCI diagnosis; patients' diagnosis confirmed by neurologist, psychiatrist, and neuropsychologist, patients' diagnosis according to the previous study	other psychiatric or neurological diseases, cancer, vascular diseases, diseases with clinically significant hepatic, pulmonary, renal, metabolic, or endocrine disturbances, inflammation, significant small vessel cerebrovascular disease, large vessel strokes	NR
McNicholas et al., 2022	all: aged over 55 years; AD: AD diagnosis based on the NIA-AA criteria; MCI: MCI diagnosis based on the NIA-AA criteria; as described by François et al., 2021	cognitive co-morbidities including PD, head trauma, learning disability, alcoholism	NR
Pekeles et al., 2019	HC: aged at least 60 years, MoCA score at least 25; AD: AD diagnosis based on the NIA-AA criteria, MCI: subjective memory complaints, normal activities of daily living and general cognitive function, objective evidence of memory impairment on testing, not demented; other disorders diagnosed according to appropriate criteria	"mixed" pathology, mixed AD/PD diagnosis	NR
Peña-Bautista et al., 2019	age 50-75 years old, AD group: positive biomarkers (neuroimaging, CSF β-amyloid, CSF tau); other participants: negative biomarkers; AD diagnosis according to the NIA-AA criteria	participants with ambiguous findings	NR
Peña-Bautista et al., 2020	AD group: altered neuropsychological evaluation (CDR > 0, RBANS.DM< 65, MMSE< 27), positive CSF biomarkers, MCI-AD: cognitive complaints without daily living activities impairment, mild AD: minor daily living activities impairment, examination according to the NIA-AA criteria	history of structural brain disease (tumour, stroke, etc.), epilepsy, major head trauma, multiple sclerosis, major psychiatric disorders, inability to undergo neuropsychological evaluations	NR

Pukhalskaia et al., 2020	HC: clinical, laboratory, and instrumental examination according to the age norm; AD: mild or moderate AD stage according to the NINCDS-ADRDA or ICD-10 criteria	HC: exacerbation of neural, cardiovascular, respiratory, and endocrine system diseases	NR
Ryu et al., 2023	AD: AD diagnosis based on the NINCDS-ADRDA criteria, MMSE score, PET examination, HC: not meeting these criteria	NR	NR
Sabaei et al., 2023	HC: MMSE score equal to or more than 26, no history of neurological diseases; AD: AD diagnosis according to the NIA-AA criteria, no other diseases that affect cognition; PD: PD diagnosis according to appropriate criteria	AD, PD: drugs other than medications for treating the underlying neurological disease; all participants: history of vascular dementia, head injury, stroke, liver or kidney failure, other dementias	NR
Shi et al., 2011	HC: MMSE score equal to or more than 27, paragraph recall scores >6; AD: AD diagnosis according to the NINCDS-ADRDA criteria	HC: history of neurological disease, evidence of cognitive or functional decline	NR
Tvarijonaviciute et al., 2020	aged 65 years or older; AD: confirmed diagnosis of AD dementia; HC: functionally and socially active, MMSE score equal to or more than 28	All: chemotherapy, radiotherapy of the head and neck, serious personality disorders, schizophrenia, epilepsy, drug abuse, meningioma other brain tumor, chronic treatment with cyclosporine or other immunosuppressor, uncontrolled acute or chronic disease; HC: dementia, cognitive impairment, neurodegenerative disease	NR
Zalewska et al., 2021	AD: AD diagnosis according to the NINCDS-ADRDA criteria; HC: no neurological diseases or cognitive impairment, attending dental check-ups, matching to the study group in terms of gender and age	history of hemorrhagic, ischemic stroke, traumas within the cerebral part of the skull, severe depression, vascular dementia, tumors within the brain, diabetes, insulin resistance, thyroid diseases, obesity, psoriasis, PD, Huntington's disease, deficiency of vitamin B12 or folic acid, abuse of smoke cigarettes or alcohol, antibiotics or supplements during the 6 months preceding material collection, change of diet	non-smokers

Legend: AD, Alzheimer's Disease; A-T-N, Amyloid, Tau, and Neurodegeneration classification; CCMD-3, Chinese Classification of Mental Disorders, Third Edition; CDR, Clinical Dementia Rating; CSF, cerebrospinal fluid; DM scale, Dementia-Metabolic; DSM-III-r, Diagnostic and Statistical Manual of Mental Disorders, Third Edition, revised; DSM-IV, Diagnostic and Statistical Manual of Mental Disorders, Fourth Edition; HC, healthy controls; ICD-10, International Classification of Diseases; MCI, Mild Cognitive Impairment; MMSE score, Mini-Mental State Examination; MoCA score, Montreal Cognitive Assessment; NIA-AA criteria, National Institute on Aging and Alzheimer's Association; NINCDS-ADRDA criteria, National Institute of Neurological and Communicative Disorders and Stroke and the Alzheimer's Disease and Related Disorders Association criteria; NR, not reported; PD, Parkinson's Disease; PET, Positron Emission Tomography; RBANS scale, Repeatable Battery for the Assessment of Neuropsychological Status; UPSIT, University of Pennsylvania Smell Identification Test.
